# Cholesterol and Albumin as Key Modulators of ICG Photostability in Aqueous Solution

**DOI:** 10.3390/molecules31152738

**Published:** 2026-08-06

**Authors:** Wiktoria Mytych, Mohammad A. Saad, Dorota Bartusik-Aebisher, David Aebisher, Gabriela Henrykowska

**Affiliations:** 1 English Division Science Club, Faculty of Medicine, Collegium Medicum, University of Rzeszów, 35-310 Rzeszów, Poland; wm108056@stud.ur.edu.pl; 2 Wellman Center for Photomedicine, Massachusetts General Hospital, Harvard Medical School, Boston, MA 02114, USA; msaad1@mgh.harvard.edu; 3 Department of Biochemistry and General Chemistry, Faculty of Medicine, Collegium Medicum, University of Rzeszów, 35-310 Rzeszów, Poland; dbartusikaebisher@ur.edu.pl; 4 Department of Photomedicine and Physical Chemistry, Faculty of Medicine, Collegium Medicum, University of Rzeszów, 35-310 Rzeszów, Poland; 5 Department of Epidemiology and Public Health, Faculty of Medicine, Medical University of Lodz, Tadeusza Kosciuszki 4, 90-419 Lodz, Poland

**Keywords:** indocyanine green, ICG, photostability, photodegradation, human serum albumin (HSA), cholesterol, near-infrared fluorescence, NIR

## Abstract

Indocyanine green (ICG) is a clinically approved near-infrared fluorescent dye used in medical imaging and diagnostics but has limitations owing to its poor photostability in aqueous environments. This paper has explored the role of human serum albumin (HSA) and cholesterol in protecting ICG photostability. Pure ICG, an HSA-ICG complex, and an ICG–cholesterol colloidal assembly solution were stirred in the dark (control) and under continuous broadband irradiation (400–1600 nm, approximately 1.4 W), and absorption spectra (550–950 nm) were taken every 1 min, over 15 min. All the formulations were stable in the dark, with minimal total variance in absorbance. Pure ICG significantly photodegraded under irradiation (55 ± 2.75–65 ± 3.25% loss of maximum absorbance). The introduction of HSA and cholesterol limited the photodegradation, resulting in 15 ± 0.75–30 ± 1.5% and 25 ± 1.25% losses in maximum absorbance, respectively, upon irradiation. The modulators produced a significant increment in initial NIR absorbance (*p* < 0.001) and retained significantly high stability during irradiation (*p* < 0.01). Moreover, both modulators reduced photooxidative damage, as shown by the lower level of singlet oxygen (^1^O_2_) generation in the presence of HSA-ICG (35 ± 1.75%) and ICG–cholesterol (19 ± 0.95%) compared to pure ICG (57 ± 2.85% after 15 min). These results reveal that cholesterol is the best stabilizer of ICG photostability. By safely dissipating excitation energy via non-radiative decay, cholesterol demonstrates strong potential for enhancing ICG performance in photothermal therapy (PTT), whereas HSA remains the optimal modulator for near-infrared fluorescence imaging and photodynamic therapy.

## 1. Introduction

Indocyanine green (ICG) is a non-radioactive, near-infrared fluorescent dye that is currently used in medical imaging and diagnostics due to its favorable safety profile. Although approved by the FDA, ICG faces limitations in aqueous conditions, where it is rapidly degraded depending on light exposure, temperature, and solvent composition [1,2]. Previous research has revealed that the decomposition of ICG in water is first order, with the rate accelerating in the presence of light and high temperatures, leading to degradation products like leucoforms, thereby reducing fluorescence efficiency [2]. The photodegradation of ICG when present in a monomeric or dimeric state in aqueous solutions is approximately 10^−3^, indicating its instability. This can be reduced in organic solvents or plasma, though it remains a problem in dilute water-based systems [1,3].

In addition to the above-mentioned stability issues, ICG has a concentration-dependent aggregation behavior, with high concentrations facilitating the formation of aggregates, changing its absorption spectrum and leading to self-quenching, which reduces the fluorescence intensity. This might not align with Lambert–Beer’s law at high concentrations of over 15 mg/L in plasma, thus limiting its use for quantification purposes [4]. This has been made worse by poor photostability in aqueous solutions at physiological temperatures, where fluorescence is rapidly lost following exposure to near-infrared light, further limiting its use in long-term imaging or therapeutic regimens such as photothermal therapy [1,3]. To address these challenges, stabilizers like albumin have been explored.

### 1.1. Albumin

Albumin, specifically human serum albumin (HSA), is an important molecule in the stabilization of ICG. This stabilization is achieved through a classic “host–guest” complexation strategy, a method widely used to adjust the physicochemical properties of pharmaceutical molecules. Through this non-covalent interaction, the host macromolecule overcomes key constraints of the free guest indocyanine green, mainly self-quenching and a poor circulation lifetime [5]. In non-covalent binding of ICG to HSA, there is an increase in the fluorescence intensity in the near-infrared region since the protein environment eliminates quenching effects that are present in aqueous conditions due to H-aggregate formation. Binding to HSA limits the molecular motion of ICG, avoiding self-quenching and enhancing its photostability, and it has been demonstrated that HSA pockets inhibit torsional motions and suppress the formation of triplet states [6]. This non-covalent interaction permits ICG to bind to HSA as a pseudofluorogenic label, making it easy to be detected using methods such as capillary electrophoresis with laser-induced fluorescence [7].

This complexation increases the in vivo half-life of ICG by decreasing its clearance rate in vivo because the larger molecular weight of the ICG-HSA complex (thought to be approximately 67 kDa) lowers the vascular permeability and increases its retention in the blood compared to free ICG [8]. This is especially useful in practices such as fluorescence-guided surgery where increased circulation time enhances imaging contrast and targeting efficacy. Furthermore, hybrid nanoparticles with albumin introduce hydrophobic pockets, which enhance those effects by promoting chemical and physical stability, resulting in enhanced aqueous stability and a reduction in degradation [9]. In general, it is evident that avoiding ICG aggregation, which results in quenching, by the use of albumin is not the only benefit, as it also improves pharmacokinetics, enabling the use of ICG in diagnostic and therapeutic applications [10]. Apart from albumin, cholesterol serves as another key stabilizer.

### 1.2. Cholesterol

Cholesterol is a major modulator in lipid bilayers, where the stability and retention of ICG in liposomal or membrane vesicles is promoted by cholesterol based on the fluidity of the bilayer and molecular packing. The integration of cholesterol in liposomal preparations decreases the fluidity of the membrane to form a more structured and packed liposomal preparation, which prevents the premature release of ICG, especially in conditions that require stimulation by light [11,12]. This modulation enhances the chemical stability and fluorescence of ICG, whereby lipid-bound ICG in cholesterol-containing liposomes experiences less quenching following light exposure and higher storage capacity [13]. Through integration into the phospholipid bilayer, cholesterol causes further ICG lipid affinity, especially in comparison to the free or esterified cholesterol, but overall it improves the membrane integrity against photothermal stress [14].

Moreover, in therapeutic contexts, liposomes with cholesterol stabilizers are used to inhibit the early release of ICG prior to controlled heating of the system through near-infrared emission [15]. Moreover, the presence of cholesterol in hybrid nanoparticles or liposomes prevents the spontaneous transfer of ICG to albumin because encapsulation prevents light-induced instability. This is essential in in vivo imaging because increased retention results in improved biodistribution and decreased off-target effects, and therefore cholesterol is a crucial element in optimizing delivery systems containing ICG [16]. The main biochemical properties of ICG include its high affinity for albumin, 98–99% in human plasma, and cholesterol-rich lipoproteins (HDL > LDL), which leads to significant spectral changes. These include changes in the absorption maximum (from approximately 780 nm for free, monomeric ICG in aqueous solutions to around 805–810 nm for ICG bound to proteins and lipids), increased absorption intensity, and significant molecular stabilization (lower photochemical depolarizations) [17]. These properties are still used in in vivo imaging (near-infrared fluorescence, NIRF) and ex vivo spectrophotometric analyses [18]. ICG has been shown to be selective in localizing to atherosclerotic plaques in animal models of atherosclerosis and in patients undergoing endovascular procedures [19]. The hypothesized mechanisms for this accumulation include (1) increased endothelial permeability in inflammatory areas (enhanced permeability and retention effect (EPR)), (2) adhesion to oxidized lipoproteins and macrophages within the atherosclerotic plaque, and (3) uptake by foam cells with scavenger receptors [20]. Hybrid methods have been used to validate clinical studies of NIRF-IVUS and NIRF-OCT [21]. ICG signal intensity is found to be associated with lipid content, inflammation severity, and plaque fragility.

Building on those these properties, the aim of this study was to evaluate changes in ICG absorption spectra within 15 min after exposure to visible light and to assess singlet oxygen (^1^O_2_) generation as a marker of photooxidative mechanisms. Furthermore, another important aspect of our analysis was evaluating the change in the absorption maxima of ICG, a potential photothermal and photosensitizing agent, after its incubation with cholesterol and albumin.

## 2. Results

### 2.1. Absorption Spectra of ICG

Under control conditions without irradiation, the absorption spectra of pure ICG remained unchanged throughout the 15 min measurement period. All curves overlapped, and the absorption maximum remained stable around 780–785 nm, with a peak value of 0.41–0.43 a.u. The peak shape was narrow, symmetrical, and typical of the monomeric form of the dye in aqueous solution. The lack of any noticeable intensity change, maximum shift, or band broadening clearly indicates that ICG exhibits excellent chemical and photophysical stability in the dark, with neither thermal or hydrolytic degradation nor spontaneous aggregation under these conditions. However, after 15 min of irradiation (400–1600 nm, ~1.4 W), significant photodegradation was observed. The absorbance at the maximum decreased from the initial 0.41–0.43 a.u. to only 0.12–0.18 a.u., corresponding to a loss of 55 ± 2.75–65 ± 3.25% of the initial value. The peak became visibly flattened, significantly broadened, and its maximum shifted slightly toward longer wavelengths (to ~790–800 nm). The short- and long-wavelength arms lost intensity, indicating significant photooxidative decomposition of the monomeric form of ICG. Driven by the generated singlet oxygen, the primary degradation mechanism is the oxidative cleavage of ICG’s central polymethine chain. This cleavage severs the extensive conjugated π-electron system responsible for the dye’s near-infrared absorption, breaking the molecule into smaller carbonyl-containing structural fragments such as the substituted oxoindoline derivatives and aldehydes. Because these fragmented degradation products lack the required conjugated double-bond network, they act as leucoforms with practically zero absorption capacity in the near-infrared range. These results clearly confirm that the main and almost exclusive destructive factor for pure ICG is exposure to light, whereas, in the absence of light, the dye remains stable for at least 15 min.

### 2.2. Absorption Spectra of ICG with Albumin

**Figure 1:**
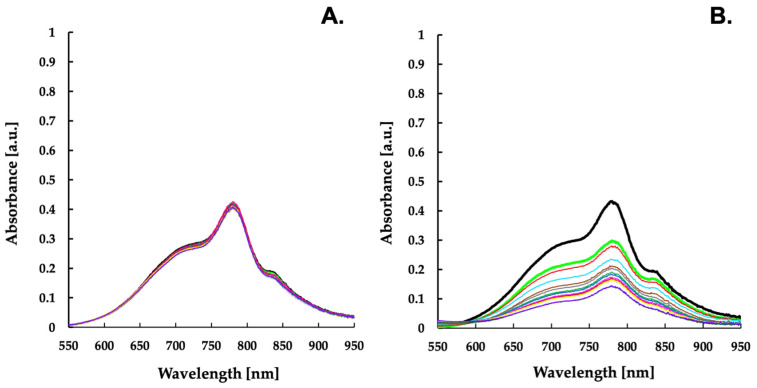
Absorption spectra of pure ICG (20 μM ICG) over a 15 min period. (**A**). Control measurements taken every 1 min in complete darkness. (**B**). Measurements taken every 1 min during continuous broadband irradiation (400–1600 nm, ~1.4 W). Colored line captions: • 1 min, • 2 min, • 3 min, • 4 min, • 5 min, • 6 min, • 7 min, • 8 min, • 9 min, • 10 min, • 11 min, • 12 min, • 13 min, • 14 min, • 15 min. Curves represent the mean of three independent experiments (n = 3). Error bars have been ommited for visual clarity.

Under control conditions without illumination (Figure 1A), the absorption spectra of the HSA-ICG complex remained stable throughout the 15 min measurement period. All curves, representing different exposure times, overlapped, and the absorption maximum remained consistently around 780–785 nm, with a peak value of 0.55–0.60 au. The peak was characterized by a shoulder at short wavelengths (~700–750 nm) and was in a narrow, symmetrical shape. No decrease in intensity, max shift, and broadening of the band was observed, suggesting that the HSA-ICG complex is chemically and photophysically stable in the dark and that there was no spontaneously degradation, hydrolysis, or aggregation in dark conditions. Moderate photodegradation occurred after 15 min (400–1600 nm, ~1.4 W) (Figure 1B). The curves clearly diverged, but the intensity drop was relatively small compared to pure ICG. The highest curves peaked at 0.40–0.52 au (a 15 ± 0.75–25 ± 1.25% decrease from baseline) and declined to 0.20–0.35 au. All curves showed a slight shift in the maximum to longer wavelengths (~790–805 nm) and a slight broadening of the peak—characteristic of partial decomposition and the formation of J-aggregates. The average absorbance loss was 15 ± 0.75–30 ± 1.5%, and the peak was only partially flattened and shifted. These results provide clear evidence of a protective effect of albumin.

### 2.3. Absorption Spectra of ICG with Cholesterol

**Figure 2:**
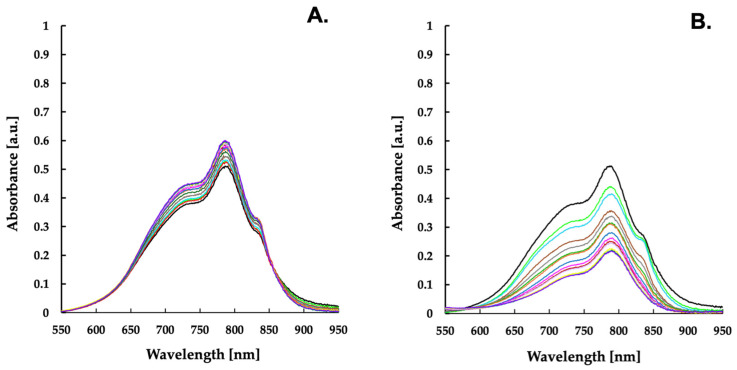
Absorption spectra of the HSA-ICG complex (20 μM ICG) over a 15 min period. (**A**) Control measurements taken every 1 min in complete darkness. (**B**) Measurements taken every 1 min during continuous broadband irradiation (400–1600 nm, ~1.4 W). Colored line captions: • 1 min, • 2 min, • 3 min, • 4 min, • 5 min, • 6 min, • 7 min, • 8 min, • 9 min, • 10 min, • 11 min, • 12 min, • 13 min, • 14 min, • 15 min. Curves represent the mean of three independent experiments (n = 3). Error bars have been ommited for visual clarity.

In the dark (Figure 2A), the absorbance of the ICG–cholesterol solution did not change significantly within the 15 min studied. No significant changes were recorded in the maximum value of 0.47–0.50 a.u. at a wavelength of about 775–785 nm and no significant changes were observed in the spectral shape: no decrease in intensity or peak shift or expansion of the band was observed. This finding validates that the chemical and optical dark stability of this complex is high and that the complex does not exhibit spontaneous degradation or aggregation. Within the 15 min of exposure to light (Figure 2B), there was slight variation compared to the rest of the test variants. The great majority of the curves had a high absorbance at peak (0.33–0.35 a.u. on the best curves), and the mean loss at baseline was only 25 ± 1.25%. The upper limit was also relatively small and the displacement of the maximum was quite minimal. These results suggest that all of the modifications investigated, except for the incorporation of the cholesterol, displayed the most potent protection against the ICG photodegradation, actually inhibiting the primary events that degrade ICG during irradiation. The outcome outlines the specific potential of cholesterol as a stabilizer in formulations meant to be used for long-term or repeated application.

### 2.4. Changes in Absorption Spectra

**Figure 3:**
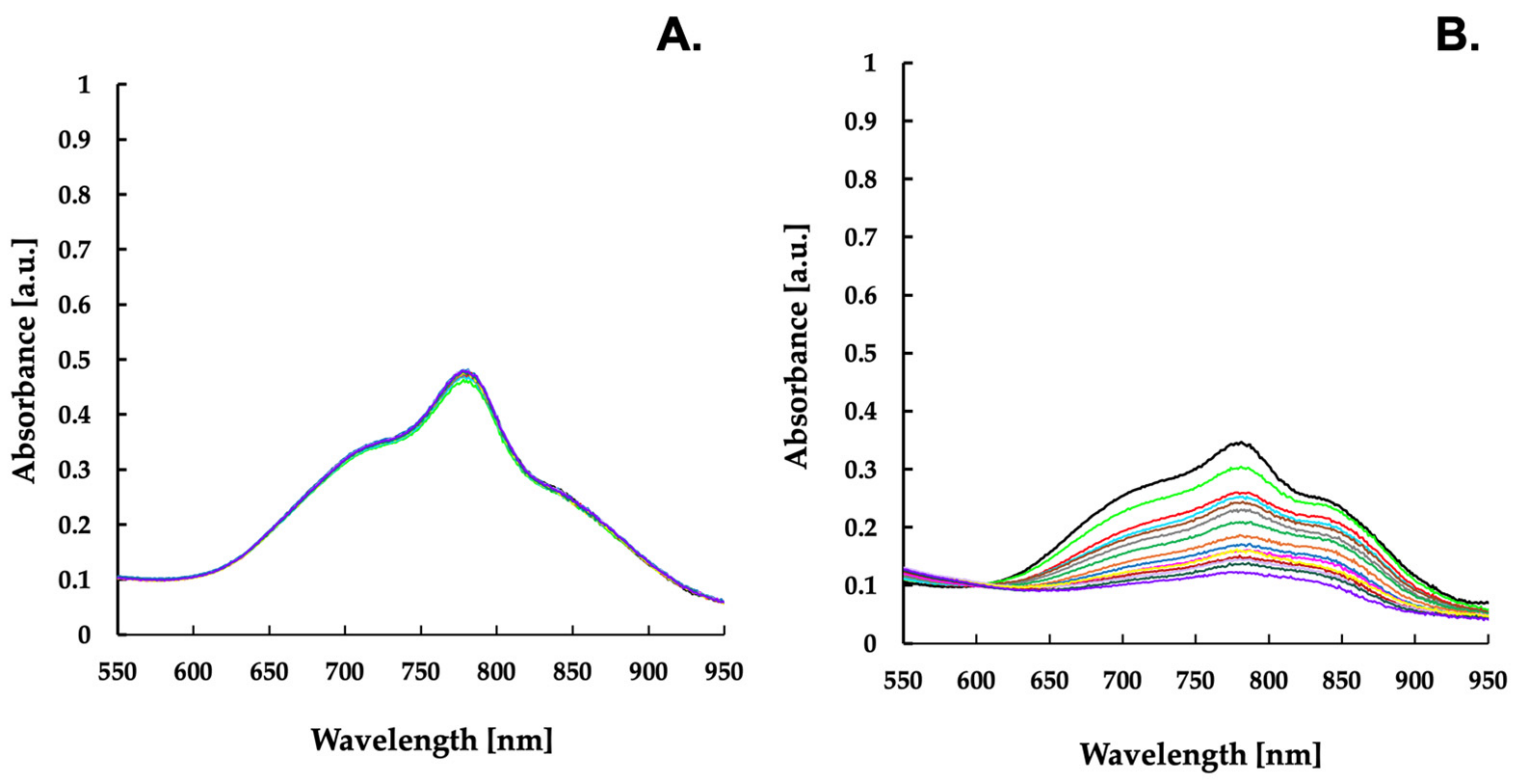
Absorption spectra of the ICG–cholesterol (20 μM ICG) colloidal assembly over a 15 min period. (**A**) Control measurements taken every 1 min in complete darkness. (**B**) Measurements taken every 1 min during continuous broadband irradiation (400–1600 nm, ~1.4 W). Colored line captions: • 1 min, • 2 min, • 3 min, • 4 min, • 5 min, • 6 min, • 7 min, • 8 min, • 9 min, • 10 min, • 11 min, • 12 min, • 13 min, • 14 min, • 15 min. Curves represent the mean of three independent experiments (n = 3). Error bars have been ommited for visual clarity.

Under control conditions in complete darkness, the absorption spectra of all tested systems—pure ICG, the HSA-ICG complex, and the ICG–cholesterol colloidal assembly—remained highly stable throughout the 15 min measurement period. In each case, the successive spectra overlapped almost perfectly, with no detectable changes in absorbance intensity, position of the absorption maximum, or peak shape. These results confirm the excellent chemical and photophysical stability of ICG in the absence of light, regardless of the formulation, indicating the lack of spontaneous thermal degradation, hydrolysis, or aggregation under the experimental conditions. In contrast, continuous broadband irradiation (400–1600 nm, ~1.4 W) induced pronounced differences in photostability among the formulations. Pure ICG (20 μM) exhibited severe photodegradation (Figure 3B). The absorbance at the maximum decreased dramatically from the initial 0.41–0.43 a.u. to 0.12–0.18 a.u. after 15 min, corresponding to a loss of 55 ± 2.75–65 ± 3.25% of the initial value. This was accompanied by significant flattening and broadening of the peak, as well as a slight bathochromic shift in the absorption maximum (from ~780–785 nm to ~790–800 nm). Such spectral changes reflect extensive photooxidative decomposition of the monomeric form of ICG, primarily through singlet oxygen-mediated cleavage of the central polymethine chain, resulting in the formation of non-absorbing or weakly absorbing degradation products. Complexation with human serum albumin (HSA) provided substantial protection against photodegradation (Figure 3B). Although the spectra diverged over time, the decrease in absorbance was markedly lower than that observed for pure ICG. The maximum absorbance dropped from the initial 0.55–0.60 a.u. by only 15 ± 0.75–30 ± 1.5% (average 20 ± 1–25 ± 1.25%), with the highest curves retaining 0.40–0.52 a.u. after 15 min of irradiation. The changes were limited to a moderate broadening of the peak and a slight red shift (~790–805 nm), consistent with the partial decomposition and limited formation of J-aggregates. These findings clearly demonstrate the protective effect of albumin, which restricts the mobility of the ICG molecule, reduces the generation of triplet states, and thereby inhibits photochemical degradation pathways. The most effective stabilization was achieved with the ICG–cholesterol colloidal assembly. Under irradiation, this formulation exhibited the smallest changes among all tested systems. The absorbance at the peak decreased by a mean of 25 ± 1.25%, while the absorption maximum remained relatively stable with only a minimal shift. The peak shape was largely preserved, showing limited broadening and maintaining a relatively narrow profile. These results indicate that cholesterol exerts the strongest photoprotective effect, most likely by creating a lipophilic microenvironment that efficiently suppresses the primary events leading to ICG photooxidation. Taken together, the absorption spectroscopy data highlight the critical role of formulation components in modulating the photostability of ICG. While pure ICG undergoes rapid and extensive photodegradation, both HSA and, particularly, cholesterol significantly enhance the dye’s resistance to light-induced decomposition, making them promising excipients for the development of more stable ICG-based diagnostic and therapeutic preparations.

### 2.5. Singlet Oxygen Detection Results

**Figure 4:**
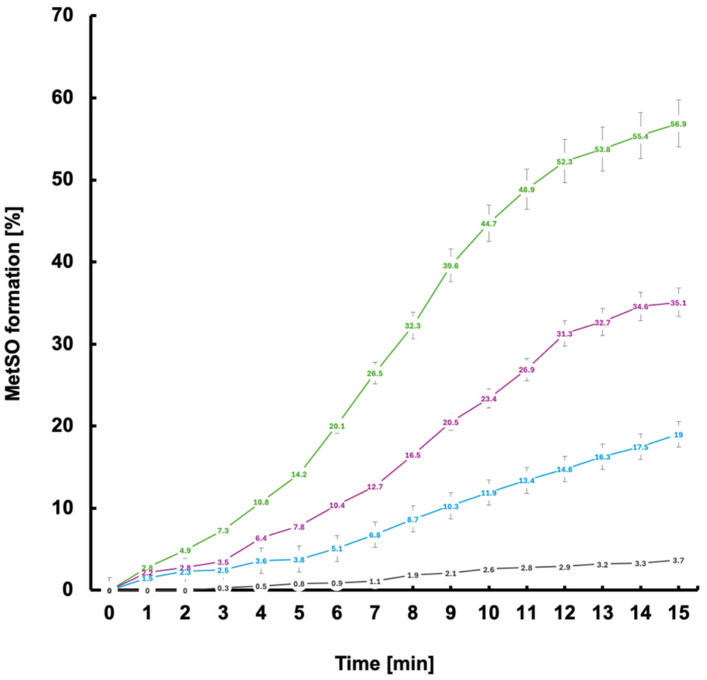
Methionine sulfoxide (MetO) formation as a function of irradiation time (0–15 min) for pure ICG, the ICG–cholesterol colloidal assembly, and the HSA-ICG complex. Data points represent the mean of three independent experiments (n = 3) and error bars represent the standard deviation. The curves illustrate the rate of methionine oxidation by singlet oxygen (^1^O_2_) generated during irradiation. Captions: • ICG, • ICG–cholesterol colloidal assembly, • HSA-ICG. The control (methionine without ICG) is shown by a gray line with a slope close to zero.

The generation of singlet oxygen (^1^O_2_), tracked indirectly via methionine oxidation to MetO, varied significantly among the microenvironments during the 15 min of irradiation. Pure ICG exhibited intense, continuous photodynamic activity, oxidizing approximately 57 ± 2.85% of the available methionine. In the presence of albumin, this photooxidative activity was significantly attenuated; the MetO level reached only 35 ± 1.75%, representing a 39 ± 1.95% reduction in effective ^1^O_2_ production compared to the pure dye. This indicates that HSA effectively restricts ^1^O_2_ diffusion or partially scavenges reactive oxygen species. The ICG–cholesterol colloidal assembly demonstrated the strongest suppression of photodynamic activity, yielding only 19 ± 0.95% MetO formation (a 67 ± 3.35% reduction compared to pure ICG). This profound limitation of ^1^O_2_ availability confirms that the lipophilic cholesterol microenvironment severely impedes the photooxidative cascade, strengthening its role as a highly effective photostabilizer. The observed differences in final oxidation states confirm that the use of ICG in these formulations can very effectively modulate the generation and availability of singlet oxygen, which is important for optimizing its applications in photothermal and photodynamic therapies (Figure 4).

### 2.6. Fluorescence Result

**Figure 5:**
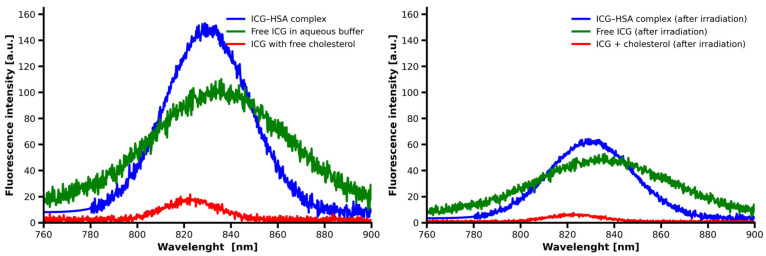
Fluorescence emission spectra of ICG in different microenvironments (at a standardized 20 μM ICG concentration) before and after irradiation. • ICG–HSA complex (peak at ≈830 nm, highest intensity); • free ICG in aqueous buffer (broad band, moderate intensity); • ICG with free cholesterol (strongly quenched, residual peak ≈ 820–825 nm). The spectra illustrate the crucial role of protein binding in the behavior of ICG fluorescence. The spectra demonstrate the photostability and microenvironment-dependent behavior of ICG upon irradiation. Curves represent the mean of three independent experiments (n = 3). Error bars have been ommited for visual clarity.

Figure 5 shows the fluorescence emission spectrum of the ICG at 780 nm under constant excitation; it can be seen that the three environments under study exhibited distinct differences that were characterized mainly by the difference in the ability of photobleaching with increased duration of excitation. The ICG-HSA complex exhibited the greatest fluorescence: the maximum was around 136 a.u., and the emission peak was very sharp and symmetrical, with its center being 830–832 nm. Such a red-shifted, pronounced, and sharp band is typical of monomerizing ICG in strong hydrophobic albumin aggregates, which greatly enhances quantum yield and shields the dye against aggregation and photochemical degradation. Conversely, when the same solution was placed in aqueous buffer, the fluorescence (maximum ≈ 82 a.u.) was much smaller, and the emission band was wide and weak, measuring ≈825–840 nm. The diffuse form and lower signal strength in the results are evidence of the creation of nonfluorescent H-type aggregates, as the photodegradation of the aggregates proceeded progressively during the measurement. This aggregation-induced spectral broadening in aqueous environments contrasts sharply with the behavior of ICG in organic solvents, where the dye is known to remain strictly monomeric and highly fluorescent, further validating the necessity of protective macromolecular carriers in water-based systems. The lowest fluorescence intensity was observed in the ICG–cholesterol formulation, where the emission maximum reached only 10 a.u. However, it is crucial to conceptually separate this fluoresence quenching from photochemical bleaching. This extreme quenching does not indicate dye degradation; rather, it reflects the tight molecular packing and H-aggregate formation of ICG within the lipophilic cholesterol colloidal assemblies. In this highly restricted environment, the excited dye molecules rapidly dissipate their absorbed energy via non-radiative decay pathways (heat) instead of radiative decay (fluorescence). This highly efficient non-radiative relaxation effectively outcompetes intersystem crossing to the triplet state. Consequently, the generation of singlet oxygen is severely suppressed (as corroborated by the MetO assay results). Therefore, the severe fluoresence quenching observed in the cholesterol system directly correlates with its superior chemical photostability, as it safely dumps excited-state energy as heat. The cholesterol assembly prevents the photooxidative cascade that would otherwise bleach the dye.

## 3. Discussion

The results of this study confirm that both cholesterol and albumin significantly increase the absorbance of ICG and mitigate its photodegradation upon exposure to light, with cholesterol demonstrating the strongest stabilizing effect [22,23,24]. These observations are consistent with the well-documented biochemical properties of ICG, which include a high affinity for albumin (98–99% binding in human plasma) and cholesterol-rich lipoproteins (HDL > LDL) [4,25,26]. This binding produces characteristic spectral changes, including a shift in the absorption maximum from ~780 nm (for free ICG) to 805–810 nm, increased absorbance intensity, and enhanced molecular stability [27,28,29]. Such changes were observed in clinical studies, where the incubation of ICG with serum from patients with hypercholesterolemia and suspected atherosclerosis resulted in a greater increase in signal intensity at 805 nm compared to healthy individuals (*p* < 0.001) [17,18]. This effect correlated positively with total cholesterol (r = 0.82), LDL (r = 0.78), and triglyceride (r = 0.65) levels, and negatively with HDL (r = −0.51). Mechanistically, increased ICG binding in dyslipidemia results from the increased availability of binding sites on oxidized or modified lipoproteins, which exhibit a higher affinity for the dye [20,26]. Hypoalbuminemia, often accompanying inflammation, may paradoxically increase the free ICG fraction [21]. However, in conditions of hypercholesterolemia, the dominant lipid effect suggests a predominance of interactions with lipoproteins [22,30].

This phenomenon reflects the pathophysiology of atherosclerosis, in which oxidized LDLs are internalized by macrophages via scavenger receptors, leading to the formation of foam cells, oxidative stress and plaque development [1,31,32]. It is important to note the specific mechanics of the light-induced degradation observed in our broadband (400–1600 nm, ~1.4 W) experimental setup. In accordance with the first law of photochemistry, photochemical degradation is driven exclusively by the photons absorbed by the target molecule. Therefore, the photodegradation of ICG and its complexes is primarily mediated by a small fraction of the total emitted power, specifically the near-infrared (NIR) band (approximately 700–850 nm) that directly overlaps with the absorption spectrum of the dye. Ideally, assessing the absolute photostability would involve calculating the quantum yield of photodegradation (ϕ_deg_), defined as

ϕdeg=numberofmoleculesdegraded/numberofphotonsabsorbed

However, calculating the exact portion of light absorbed and determining the absolute ϕ_deg_ require monochromatic excitation (e.g., laser sources) and precise actinometry. Because a continuous broadband source was used, we can only evaluate the relative photodegradation rates among the formulations. The significant reduction in absorbance loss demonstrates that both HSA and cholesterol drastically lower the relative ϕ_deg_ compared to pure ICG, but exact quantitative estimates remain a limitation of the current experimental design.

Photons outside this specific band, such as those in the visible spectrum (400–700 nm) and deeper infrared (>850 nm), are not significantly absorbed by the ICG molecules and thus do not directly participate in the excitation-state chemistry or subsequent photooxidative damage. While deep infrared wavelengths can be absorbed by the aqueous solvent to generate localized heat (which can indirectly accelerate thermal degradation), our experimental setup actively controlled the temperature to fluctuate no more than 2 degrees Celsius, isolating the observed degradation to purely photochemical pathways driven by the NIR band. Furthermore, the possibility that these added modulators simply act as physical optical filters can be ruled out. Both human serum albumin and cholesterol absorb light exclusively in the ultraviolet region (<300 nm) and are strictly optically transparent in the near-infrared window (700–850 nm). Because these modulators do not attenuate the specific NIR wavelengths responsible for driving ICG photooxidation, their potent protective effects must be attributed entirely to functional molecular and supramolecular interactions. By encapsulating the dye, these matrices structurally restrict the torsional molecular motions of ICG, suppress the formation of triplet states and severely impede the localized diffusion of reactive oxygen species rather than passively blocking the incident light.

### 3.1. Role of Albumin

The molecular insights into the associations of ICG with albumin and HSA include a robust host–guest relationship. The binding within the hydrophobic pockets (site IIA and site IIIA of Sudlow) of the host protein limits the molecular movements of the guest ICG molecule, leading to an improvement of its photophysical characteristics [33]. The sites are in subdomains of HSA and create an environment that is hydrophobic, and thus the amphiphilic structure of the ICG is recognized and binds non-covalently through hydrophobic and electrostatic forces [34]. This localization suppresses torsional movements in the excitation state of ICG, which decreases non-radiative decay and the transition to the triplet state, enhancing fluorescence quantum yield and photostability [6]. The use of the shaped femtosecond laser pulses has shown that the HSA pocket changes the dynamics of ICG, and low-frequency vibrational modes become more dominant in the bound form. ICG is present in the binding stoichiometry, usually at 1:1 or more, and the resolution of free and bound states is confirmed by capillary electrophoresis [35,36]. These pockets also enhance the stability of ICG in hybrid systems, and the complex can be utilized in imaging—for example, in targeted tumor imaging of SPARC-expressing glioblastomas.

### 3.2. Role of Cholesterol

Cholesterol alters the molecular assembly of ICG-loaded liposomal bilayers by increasing the membrane order and decreasing fluidity. In this supramolecular host–guest architecture, the cholesterol-modulated lipid environment acts as a robust host matrix. This matrix consequently stabilizes the guest ICG and prevents photobleaching [37]. Cholesterol intercalates between phospholipid chains, condensing the bilayer, increasing the packing density, and reducing defects that may cause the leakage of ICG or its exposure to degradative factors. This mechanical reinforcement decreases alterations in permeability using light, maintains the integrity of ICG under near-infrared radiation exposure, and avoids photodegradation [38]. In related systems, the presence of cholesterol also suppresses photobleaching by inhibiting the diffusion of oxygen through the bilayer, since, in studies with lipid-bound ICG, reduced fluorescence quenching has been observed [39]. In general, this modulation facilitates efficient photothermal therapy (PTT) and imaging since it maintains long-lasting ICG retention and immunity to environmental stress.

When combining these modulators, ICG–albumin–cholesterol complexes exhibit better photophysical characteristics than free ICG, with a bathochromic shift (red shift) in the absorption spectra as well as a much higher fluorescence intensity [40,41]. The spectral change (ca. 25 nm) of the extended wavelengths and loss of cholesterol-sensitive conformational motions can be attributed to a change in the solvent and protein–lipid in the protein–lipid interactions [41]. This result enables treatment and imaging at higher wavelengths with higher penetration capabilities, while the complexation decreases self-quenching and increases fluorescence intensity. As an example, ICG-HSA complexes become strong enough to be fluorescent in dilute solutions in which free ICG is weakly fluorescent [8]. Packing in bilayers further increases fluorescence intensity in liposomal systems containing cholesterol, and studies have shown that the packed bilayer can stabilize ICG against aggregation and enhance fluorescence signals up to 6 folds compared to free ICG [41]. Comparative studies show that such complexes are able to retain large quantum yields, with improvements of around 2.7% with free ICG to in excess of 16.8% with templated J-aggregates in the presence of lipids and proteins [40,42]. In general, synergistic stabilization is more effective for enhancing contrast in imaging applications. Extending these findings to in vivo applications, ICG–albumin–cholesterol complexes demonstrate tumor accumulation and a higher tumor-to-background ratio (TBR), indicating progress as of 2026 [43]. These complexes take advantage of the increased permeability and retention effect to preferentially accumulate in tumor tissues, and it has been shown in cancer model systems that the complexes have a long retention time of up to 24–48 h, whereas free ICG has a short retention time of up to 10 min [44]. In a rectal cancer model, liposomal ICG containing cholesterol showed a TBR of 4.2 ± 0.18, which is much higher than free ICG and allows for specific tumor localization, with applications in intraoperative imaging [45]. Current experiments in cancer therapy indicate TBRs of up to 6.5 in vivo, where the specificity of sentinel lymph node mapping is enhanced and off-target biodistribution is minimized [46]. Complexes demonstrate improved contrast in colorectal liver metastases, and the optimization of TBR with modified dosing reflects the potential efficacy of this method in this model of fluorescence-guided surgery. These formulations, in general, improve the therapeutic targeting of PTT and imaging, with low accumulation of the formulations in non-tumor organs within 24 h. Advancing such stability and selectivity profiles is crucial, as demonstrated by recent developments in high-contrast fluorescent probes for rapid intraoperative tumor margin identification [47].

### 3.3. Future Directions

Our research on the physicochemical properties of ICG, including its photostability, aggregation, and singlet oxygen production in the presence of albumin and cholesterol, opens the way for its practical use in the context of cardiovascular diseases such as atherosclerosis.

**Figure 6:**
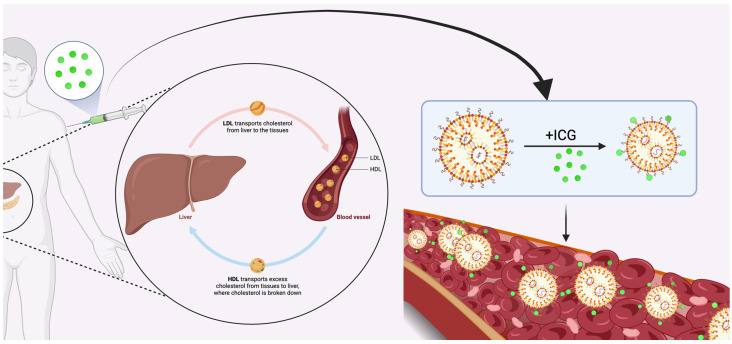
Schematic overview of lipoprotein cholesterol metabolism and the mechanisms of selective ICG accumulation in atherosclerotic plaque structures.

**Figure 7:**
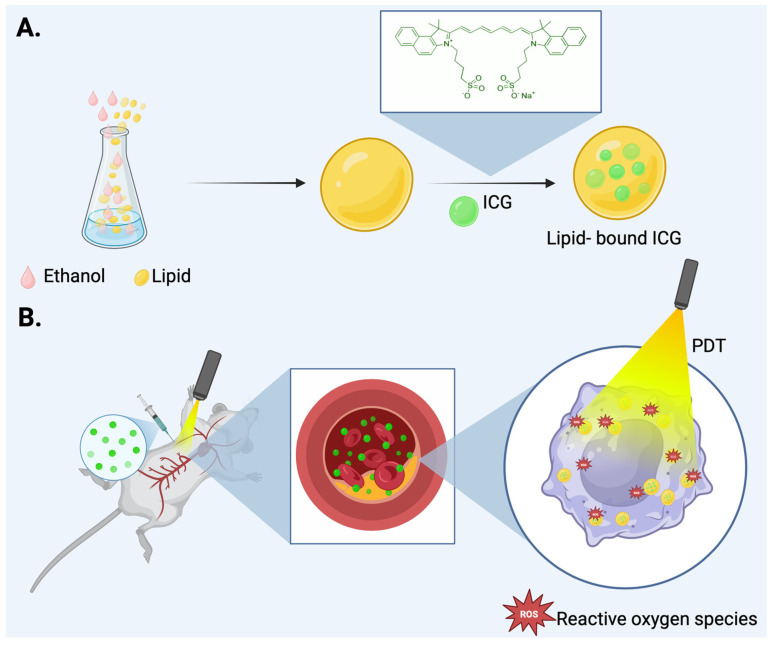
(**A**) shows the preparation of lipid nanoparticles with incorporated ICG. First, the lipids (shown as yellow beads) are dissolved in ethanol (pink droplets), after which the mixture self-assembles to form spherical liposomes or lipid nanoparticles. Free indocyanine green (green beads) is then added to the prepared structures, which spontaneously incorporates into the lipid bilayer or the interior of the nanoparticles, resulting in a lipid-bound ICG formulation. (**B**) shows the therapeutic use of lipid-bound ICG nanoparticles in an animal model of atherosclerosis. During the intravenous administration of the lipid-bound ICG nanoparticle formulation, the green beads represent these nanoparticles circulating in the blood. Due to the inherent lipophilicity of ICG and the properties of nanoparticles, the drug selectively accumulates in atherosclerotic plaques (depicted as a red circle with visible blood vessels, accumulated lipids, and red blood cells within, reflecting the lipid-rich, inflammatory environment of the plaque). A near-infrared laser beam is then directed at the atherosclerotic plaque (a yellow cone of light incident on the vessel). Photon absorption by lipid-bound ICG leads to dye excitation and ROS generation. As a result, ROS activity leads to severe oxidative stress, damaging lipids, proteins, and cells within atherosclerotic plaques (including macrophages and foam cells), leading to their destruction and potentially reducing plaque thickness. This approach is being investigated as a minimally invasive treatment for atherosclerosis, preventing intima hyperplasia and reducing accumulated lipids.

Atherosclerosis is considered one of the most common causes of morbidity and mortality worldwide, as well as a cause of cardiovascular disease, including heart attack, stroke, and peripheral artery disease [22]. The persistent deposition of lipids (primarily cholesterol), macrophages, and foam cells, fibrosis, and calcification in the vessel walls cause the proliferation of atherosclerotic plaque, thereby restricting and limiting blood flow [23]. Hypercholesterolemia exacerbates this by elevating LDL levels, damaging the endothelium and triggering inflammation [24]. The basic plasma protein albumin (constituting approximately 50–60% of total plasma protein) also plays a significant role in the clinical outcome. It plays a role in maintaining oncotic pressure and delivering endogenous and exogenous substances (fatty acids, hormones, and medications) and has antioxidant properties [25]. Hypoalbuminemia is often accompanied by inflammatory conditions such as atherosclerosis, in which endothelial permeability increases, leading to the leakage of albumin into the interstitial space [26]. A schematic overview of this lipoprotein metabolism and the selective accumulation of ICG is provided in Figure 6. Upon intravenous administration, ICG distributes throughout the circulatory system and exhibits massive binding to circulating LDL particles. This high affinity leads to the formation of ICG-LDL complexes. These complexes subsequently accumulate selectively in the subendothelial layer and within atherosclerotic plaques due to several coexisting mechanisms: increased endothelial permeability in inflammatory areas, dye adsorption on the surface of oxidized lipoproteins and receptor-mediated uptake of ICG-LDL complexes by macrophages and foam cells. Low albumin levels are an independent diagnostic risk factor that predicts cardiovascular events and an unfavorable prognosis in patients with coronary artery disease [4]. Albumin also serves to transport cholesterol in complexes with lipoproteins (HDL and LDL) and regulates its bioavailability and deposition in the vessel wall [27]. Colorimetric and enzymatic methods of albumin and cholesterol diagnosis are based on UV–Visible spectrophotometry, which is rapid, inexpensive, and commercially available. However, traditional techniques are limited to the quantification of total concentrations and do not consider the functional interactions of proteins with lipids, including their significant role in the pathogenesis of atherosclerosis [28]. This is why ICG, a near-infrared tricarbocyanine dye approved by the FDA for clinical use, is becoming a potential diagnostic method [29]. Compared to classical methods for determining albumin and cholesterol, the spectrophotometric technique with ICG provides additional functional information regarding protein–lipid interactions, which are key in the pathogenesis of atherosclerosis (Figure 7) [3].

It is rapid (analysis time < 45 min), requires a small sample volume (~100 μL), can be implemented on standard UV-Vis spectrophotometers available in most clinical laboratories, and is attractive as a screening method [48]. An additional advantage is the interference from hemoglobin, bilirubin, or triglycerides in the near-infrared range [49]. The current in vitro results are consistent with in vivo imaging studies, in which ICG selectively accumulates in lipid- and macrophage-rich atherosclerotic plaques, particularly in areas of increased endothelial permeability (EPR effect), adhesion to oxidized lipoproteins, and internalization by foam cells [17,18,50]. Hybrid techniques such as NIRF-IVUS and NIRF-OCT have confirmed the correlation of ICG signal intensity with lipid content, inflammation severity, and plaque fragility in animal models and in patients undergoing endovascular procedures [51,52]. While our purely in vitro model system with pure cholesterol and albumin is simplified, it serves as a foundational surrogate for understanding these mechanisms. With future clinical validation, similar principles might eventually aid cardiovascular risk stratification based on routinely collected serum without the need for invasive imaging [51,53]. In this context, albumin stabilizes ICG for selective plaque accumulation via ERP and macrophage binding [21,53]. HSA-ICG complexes are being investigated as nanoplatforms for PDT/PTT, improving bioavailability and efficacy in destroying inflammatory cells without toxicity to healthy tissues [52]. Liposomes or lipoproteins containing ICG and cholesterol mimic HDL, targeting lipid plaques and increasing accumulation in macrophages, ROS generation, and plaque stabilization (reducing inflammation and apoptosis of vascular smooth muscle cells (VSMCs)) [30]. While the unexposed groups were stable, the addition of albumin/cholesterol increased absorbance, improving the imaging signal and potential PDT efficacy even under low-light conditions [53]. This comparison demonstrates how albumin and cholesterol protect ICG from degradation—critical for PDT, where light is essential but causes rapid degradation of pure ICG (short half-life ~3–4 min, low photostability) [28]. With albumin or cholesterol, ICG demonstrates a longer duration of action and higher ROS production in vitro. While our simplified models suggest the potential for selective destruction of macrophages in the plaque, which could hypothetically lead to plaque stabilization and reduced restenosis, these outcomes represent extrapolations. They must be rigorously validated in future in vivo studies [52]. Cholesterol has proven to be the best stabilizer, ideal for targeting lipid plaques, allowing for optimized formulations (e.g., nano-HDL with ICG) for clinical PDT in atherosclerosis, with minimal toxicity and precise action [30]. The spectrophotometric determination of changes in ICG absorbance after incubation with serum represents a novel, scientifically valid method for assessing lipid–protein metabolism disorders in atherosclerosis. It combines simplicity and availability with high specificity for key pathophysiological processes. Future studies should consider the physicochemical optimization of ^1^O_2_ production in ICG–albumin–cholesterol systems, e.g., by measuring quantum yield in atherosclerosis models. The literature indicates that, in lipid-rich environments (such as cholesterol-rich atherosclerotic plaques), Φ_Δ can be modulated by ICG transfer between liposomes and albumin, which influences the effectiveness of PDT in reducing inflammation [54,55,56]. EPR experiments could confirm these mechanisms in vivo, enabling the development of theranostics with controlled ROS generation [57]. Future research should validate this in prospective patient cohorts while correlating with markers of inflammation and oxidation and exploring applications in therapy monitoring.

### 3.4. Limitations

The study was conducted solely in vitro under aqueous conditions, which does not fully reflect the complex physiological conditions in vivo. The results require confirmation in animal models or clinical trials. The study was limited to a 15 min exposure to broadband light, which does not allow for the assessment of long-term stability during longer imaging or therapy sessions. A broader range of concentrations is required; specifically, the quenching and stabilizing effects of cholesterol were evaluated at a single molar excess (5:1), whereas human serum albumin was utilized at 3:1 molar excess. Because the degree of photoprotection depends heavily on the stabilizer-to-dye ratio, we cannot completely rule out that the superior photostability observed in the cholesterol assembly is partially due to this higher molar excess. While this fixed ratio successfully established proof-of-concept stabilization, comprehensive dose–response and strictly equimolar comparative experiments are necessary. Future studies must investigate various ICG-to-cholesterol ratios to evaluate saturation limits, optimize quenching dynamics and establish concentration-dependent binding kinetics. The assessment of singlet oxygen was based solely on the indirect method of methionine oxidation, lacking direct measurements of other reactive oxygen species. Furthermore, while our data strongly suggests that the primary degradation mechanism is oxidative cleavage driven by ^1^O_2_, definitive proof of this hypothesis requires parallel photodegradation experiments in fully deoxygenated (e.g., argon-purged) solutions. Because these anoxic controls were not performed, we cannot definitively rule out minor contributions from non-oxidative, light-induced degradation pathways. The study compared only three formulations without extensive comparison with other stabilizers or assessment of additional photophysical parameters. Furthermore, we did not perform structural characterizations such as dynamic light scattering, zeta potential or electron microscopy on the formulations. Therefore, the exact physical dimensions and structural nature of the ICG–cholesterol colloidal assemblies remain undefined in this current scope. Additionally, while the spectral flattening indicates the formation of non-absorbing leucoforms (such as oxoindoline derivatives and aldehydes resulting from polymethine chain cleavage), we did not analytically detect these specific degradation products. Future studies must employ liquid chromatography–tandem mass spectrometry (LC-MS/MS) to isolate, identify and quantify the exact photoproducts generated in each microenvironment. Finally, the evaluation of photodynamic activity relied on an indirect methionine oxidation assay. While ICG photochemistry is primarily driven by Type II ^1^O_2_ generation, we cannot entirely rule out partial interference from other reactive oxygen species generated via Type I pathways. Further studies must employ specific ROS scavengers (e.g., sodium azide) or direct ^1^O_2_ phosphorescence detection at 1270 nm to definitely isolate the responsible oxidative species. These limitations indicate the need for further in vivo studies performed under various conditions to further confirm the clinical relevance of the results.

## 4. Materials and Methods

### 4.1. Materials

Indocyanine green (ICG) United States Pharmacopeia Reference Standard was purchased from Alchem Grupa (Toruń, Poland). Deionized water was obtained from a Reverse Osmosis Systems (Supreme, New York, NY, USA) water purification system. All materials and solvents were used as received without further purification. Aqueous solutions of ICG were prepared at a concentration of 20 μM. Cholesterol purchased from Glentham Life Sciences (Corsham, UK) was dissolved in absolute ethanol to prepare a 50 mM stock solution, which was diluted in phosphate-buffered saline (PBS) immediately before use to achieve a final concentration of 0.1 mM. Human serum albumin purchased from Merck (Darmstadt, Germany) was used at a 3:1 molar ratio (HSA: ICG).

### 4.2. Preparation of Formulations

**Figure 8:**
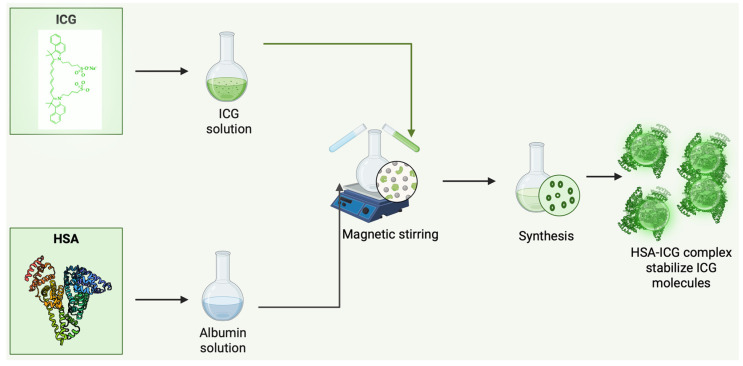
Schematic diagram showing the formation of a stable, non-covalent complex between ICG and human serum albumin (HSA) via magnetic stirring.

Aqueous ICG solutions were prepared at a concentration of 20 μM using deionized water (Figure 8). The process of forming this stable non-covalent complex is illustrated in Figure 2. For the HSA-ICG complex, HSA was added at a molar ratio of 3:1 (HSA:ICG) and vigorously mixed at room temperature in a conical flask on a magnetic stirrer to facilitate non-covalent binding. During this process, simple non-covalent mixing at room temperature allows the ICG molecules to spontaneously enter and become trapped within the hydrophobic pockets of HSA, granting the otherwise unstable dye significantly improved chemical and photophysical stability. For the ICG–cholesterol colloidal assembly, a 50 mM stock solution of cholesterol in absolute ethanol was diluted in PBS to a final concentration of 0.1 mM. Because of cholesterol’s extremely low aqueous solubility, this solvent-shifting method results in the spontaneous formation of a cholesterol colloidal suspension. This suspension was then mixed with the ICG solution under similar conditions, allowing the dye to partition into the lipophilic aggregates. This simplified binary system utilizing free cholesterol, rather than complete multi-component phospholipid liposome, was deliberately chosen to isolate the direct molecular stabilizing effects of the cholesterol molecule on ICG, stripping away the confounding physical variables of complex lipid bilayer dynamics. The molar excess of the host molecules in both formulations (3:1 excess of HSA and 5:1 excess of cholesterol relative to the 20 μM ICG) was deliberately chosen to ensure the complete non-covalent encapsulation of the dye, preventing the presence of unbound ICG and subsequent H-aggregate formation. All complexes were transferred to appropriate cuvettes for subsequent measurements.

### 4.3. Absorption Measurements

Absorption spectra were recorded using a Cary 60 UV-Vis spectrophotometer Agilent (Santa Clara, CA, USA) in the wavelength range of 550–950 nm. Sample and blank solutions were measured in Thorlabs quartz cuvettes with a capacity of 3500 μL and an optical path length of 10 mm.

### 4.4. Irradiation

**Figure 9:**
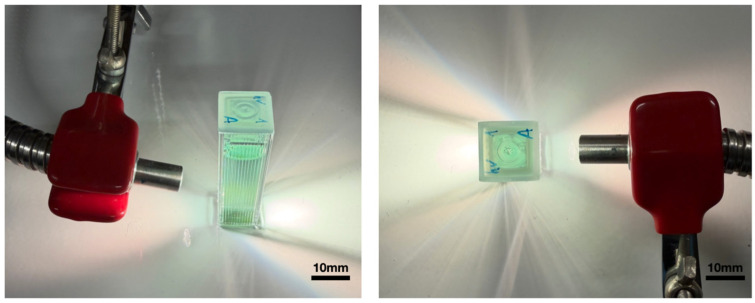
A real-life experimental setup for controlled exposure of ICG solution samples (pure, with albumin and with cholesterol) for 15 min to assess their photostability. In the center of both photographs is a standard 10 mm optical length quartz cuvette filled with a faintly greenish ICG solution. Two massive THORLABS fiber optic heads are positioned symmetrically on either side of the cuvette. Each fiber optic tip has a diameter of approximately 8–10 mm and is mounted on a laboratory stand approximately 10 mm from the cuvette wall. A very intense broadband beam of light (400–1600 nm, power approximately 1.4 W at the tip) emanates from the tips, continuously and uniformly illuminating the entire sample volume along a 10 mm optical path. Under such intense illumination, the ICG solution emits a characteristic intense green fluorescence, which is particularly visible in the right image. This creates a bright, almost blinding green glow that fills the interior of the cuvette and radiates outward as diffuse reflections. Additionally, both frames reveal numerous rainbow halos and diffraction effects around the light beam, resulting from the strong illumination of the quartz material and the dye itself. The left photo was taken from a slightly more lateral angle, allowing for a better understanding of the geometry of the entire system, the arrangement of the optical fibers, and the direction of the beam propagation.

Three samples were individually irradiated (Figure 9) for 15× intervals of 1 min each, and the absorption spectra were recorded after each minute of irradiation at 24 °C. Sample irradiation was performed using a high-intensity fiber optic illuminator with a 91 cm fiber bundle purchased from THORLABS (Newton, NJ, USA), with a power of 1.4 W at the fiber tip at maximum bulb intensity, covering a wavelength range of 400–1600 nm. We monitored the temperature of the samples during irradiation using a digital thermometer, ensuring that it did not rise above 2 °C to exclude the influence of thermal effects.

The THORLABS fiber optic illuminator head has an effective beam core diameter of 6.4 mm, with a metal ferrule of approximately 8–10 mm. The light beam is circular, conical, and has high divergence (NA ≈ 0.39–0.50) without collimation, resulting in a rapidly expanding illumination spot. The head tip is 10 mm from the front of the cuvette, providing illumination along an optical path of exactly 10 mm in a standard quartz cuvette (inner width 10 mm, outer dimensions 12.5 × 12.5 × 45 mm, volume 3.5 mL). With this close positioning, the cuvette spot is 7–15 mm in diameter, covering most of the sample volume and enabling efficient exposure of the entire optical path.

### 4.5. Singlet Oxygen Detection Using Methionine

**Figure 10:**
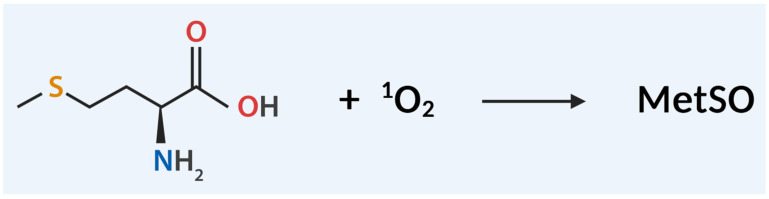
A chemical reaction in which L-methionine (Met) is oxidized by singlet oxygen (^1^O_2_), forming methionine sulfoxide (MetO). ^1^O_2_ acts as a strong, selective oxidant. This reaction is typical of photooxidation processes or other singlet oxygen-generating systems. This transformation is one of the most important post-translational oxidative modifications of proteins. Methionine residues are among the amino acids most susceptible to oxidation by singlet oxygen and other ROS.

To assess the generation of singlet oxygen (^1^O_2_) in the samples during irradiation, methionine was employed as a chemical trap. L-Methionine purchased from Sigma-Aldrich (St. Louis, MO, USA) was dissolved in PBS to prepare a 10 mM stock solution, which was added to the ICG solutions (pure ICG, HSA-ICG, ICG–cholesterol) to achieve a final concentration of 1 mM methionine in each sample. Methionine reacts selectively with ^1^O_2_ to form methionine sulfoxide (MetO), allowing for the indirect quantification of ^1^O_2_ production (Figure 10). For the evaluation of light-only-induced background oxidation, a control sample without ICG (methionine only in PBS) was included, which showed negligible MetO formation (<5% after 15 min).

Samples were prepared as described in the Materials Section and irradiated under the same conditions as for photostability assessment (15 × 1 min intervals at 24 °C using the THORLABS fiber optic illuminator, Newton, NJ, USA). Aliquots (100 μL) were withdrawn after each irradiation interval (0, 1, 2, …, 15 min) and immediately quenched by placing on ice to halt further reactions. The samples were then analyzed for methionine oxidation products. We used a Bruker Avance III 400 MHz NMR spectrometer (Ettlingen, Germany) to confirm the photooxidation reaction at 660 nm that produces reactive oxygen species (ROS). In cases of phosphorescence spectra, we used a fully automated high-performance fluorescence lifetime spectrometer FluoTime 300 EasyTau, PicoQuant (Berlin, Germany), which is set up to detect phosphorescence in steady-state and time-resolved mode. Unreacted methionine and methionine sulfoxide (MetO) were measured using high-performance liquid chromatography (HPLC) with the UV detector. A 1260 Infinity HPLC system (Santa Clara, CA, USA) combined with a C18 reverse-phase column (Zorbax Eclipse Plus C18, 4.6 × 150 mm, 5 μm particle size) was used. The mobile phase was a gradient of 0.1% trifluoroacetic acid in water (A) and acetonitrile (B), with 95% of A/5% B mixed with tube Number 01 and the remainder of A/B to 5% A/95% B over 20 min at a flow rate of 1 mL/min. Detection was done at 220 nm. Standard solutions of L-methionine in the concentration range of 0.1 to 2 mM were created using both L-methionine and methionine sulfoxide (both Sigma-Aldrich). The proportion of methionine oxidation was calculated ([MetO]/ ([Met] + [MetO]) × 100).

### 4.6. Fluorescence Measurement

The measurements were made using a fluorescence emission spectrum from an Agilent Cary Eclipse fluorescence spectrophotometer (Santa Clara, CA, USA) with a 150 W xenon flash lamp as an internal excitation source, two monochromators, which were dual Czerny–Turner monochromators, and a red-sensitive photomultiplier tube (PMT) detector. A xenon flash lamp was specifically chosen over continuous laser excitation to prevent measurement-induced photobleaching and localized heating during the spectral scans, ensuring the fragile non-protein-bound ICG variants were not artificially degraded by the instrument itself. All measurements were conducted at room temperature (≈22–24 °C) in typical 1 cm × 1 cm quartz fluorescence cuvettes that can be used for near-infrared measurements. Raw emission spectra were exported from Cary WinFLR software version 1.2 in ASCII format. Peak maxima positions, intensities (in arbitrary units), and overall spectral shapes were analyzed without additional smoothing or deconvolution. Intensity values were compared directly between systems (without applying quantum efficiency correction or internal filter correction due to the comparative nature of the study). All experiments were performed in independent triplicates (n = 3) to ensure reproducibility. In this setup, the HAS-ICG complex yields the highest fluorescence intensity (typical of monomeric ICG), free ICG yields significantly lower intensity due to partial aggregation, and lipid-bound ICG yields a broadened spectrum with typically the lowest intensity due to strong interactions with the lipid environment.

### 4.7. Statistical Analysis

Data were analyzed using one-way ANOVA for comparisons between groups at each time point, followed by Tukey’s post hoc test for multiple comparisons. Paired *t*-tests were used to assess changes over time within groups. Statistical significance was set at *p* < 0.05. Values are reported as means ± standard deviation (SD). All measurements were performed in independent triplicates (n = 3) to ensure reproducibility.

## 5. Conclusions

This study demonstrated that cholesterol and albumin significantly increase the absorbance of ICG and mitigate its photodegradation upon exposure to light, with cholesterol demonstrating the strongest stabilizing effect. The results confirm the dependence of ICG spectral changes (maximum shift to 805–810 nm and increased intensity) on interactions with hydrophobic domains of albumin and lipoproteins, particularly in hypercholesterolemia. The spectrophotometric technique with ICG provides unique functional information on lipid–protein metabolism disorders, beyond classical concentration measurements. It is rapid, inexpensive, and accessible, making it a promising screening tool for atherosclerosis risk stratification. Furthermore, the stabilization of ICG by cholesterol and albumin opens prospects for the development of advanced formulations for the targeted phototherapy (such as PTT using cholesterol or PDT using albumin) of atherosclerotic plaques with improved photostability, selective targeting, and higher efficacy, with minimal toxicity. In conclusion, cholesterol and albumin improve the diagnostic and therapeutic properties of ICG, contributing to new strategies for the prevention and treatment of cardiovascular disease. Further clinical trials are warranted.

## Data Availability

The original contributions presented in this study are included in the article. Further inquiries can be directed to the corresponding authors.

## Abbreviations

The following abbreviations are used in this manuscript:

ICG	Indocyanine green
HSA	Human serum albumin
NIR	Near-infrared
FDA	Food and Drug Administration
PBS	Phosphate-buffered saline
^1^O_2_/O_2_	Singlet oxygen
EPR	Enhanced permeability and retention
LDL	Low-density lipoprotein
HDL	High-density lipoprotein
NIRF	Near-infrared fluorescence
IVUS	Intravascular ultrasound
OCT	Optical coherence tomography
PDT	Photodynamic therapy
Met	L-Methionine
MetO	Methionine sulfoxide
